# Sex differences in location of initial diagnosis and discharge destination among medicare fee-for-service beneficiaries with post-stroke aphasia

**DOI:** 10.3389/fstro.2026.1904901

**Published:** 2026-07-29

**Authors:** Molly M. Jacobs, Charles Ellis

**Affiliations:** 1 Department of Health Services Research, Management and Policy, College of Public Health and Health Professions, University of Florida, Gainesville, FL, United States; 2 Department of Speech, Language and Hearing Sciences, College of Public Health and Health Professions, University of Florida, Gainesville, FL, United States

**Keywords:** aphasia, Medicare, rehabilitation, speech pathology, stroke

## Abstract

**Objective:**

To characterize sex differences in the clinical setting of initial aphasia diagnosis and the discharge destination of fee-for-service (FFS) Medicare beneficiaries diagnosed with post-stroke aphasia.

**Methods:**

Data for this project were obtained from 100% of FFS Medicare claims and included all beneficiaries who incurred an inpatient claim between January 2016 and October 2019 listing stroke as the primary diagnosis and had a claim listing aphasia diagnosis within 90 days of stroke.

**Results:**

Among those diagnosed with stroke (745,917), 14.5% (*N* = 107,753) were also diagnosed with aphasia within 90 days. Approximately 60.96% of men and 56.47% of women had their initial aphasia-related claim in an inpatient facility; 15.12% of men and 19.47% of women in a skilled nursing facility; and 8.44% of men and 7.03% of women in an outpatient facility. Roughly 14.99% of men and 11.50% of women with aphasia were discharged home; 25.38% of men and 31.37% of women were discharged to a skilled nursing facility; and 42.94% of men and 38.24% of women were discharged to a long-term care or rehabilitation facility (χ^2^ =904.70, *p* < 0.0001). Relative to men, women were significantly more likely to be discharged to a skilled nursing facility (OR = 1.35, 95% CI = 1.31, 1.39), but less likely to be discharged to a long-term care or rehabilitation facility (OR = 0.83, 95% CI = 0.81, 0.86).

**Discussion:**

Sex differences existed in the location of aphasia diagnosis and their destination following acute care discharge. However, the cause of these differences is unknown or how they related to quality-of-care.

## Background

In the United Stated the annual rate of stroke is approximately 795,000 people (Martin et al., 2024). Of those ~ 610000 are first stroke events and 185,000 are recurrent strokes (Martin et al., 2024). The direct and indirect costs of stroke in the US is believed to be greater than 56 billion dollars annually (Martin et al., 2024). Many stroke survivors are left with significant disability, including motor dysfunction, spasticity, cognitive impairment, dysphagia, depression, epilepsy, and aphasia (Poomalai et al., 2023). Reported post-stroke disability can result in reduced independence and poor quality of life (QOL) for many stroke survivors (Butsing et al., 2025).

There is evidence that stroke is more likely to occur in women than in men (Rexrode, 2022). The stroke literature suggests stroke occurs more often in women because women have longer life spans and other unique risks such as pregnancy, preeclampsia, and the use of oral contraceptives (Yoon and Bushnell, 2023). There is also evidence that women who experience stroke present differently than men, thereby raising concern about early stroke management (Owais et al., 2024). Similarly, women have greater disability and are more likely to report more negative post-stroke functional limitations, especially in the physical domain (Oliveira et al., 2023). Finally, women experience poorer functional recovery and lower QOL than men after stroke (Yoon and Bushnell, 2023).

Among the many post-stroke conditions is aphasia, where concerns about sex differences have emerged. Aphasia is an acquired language disorder that often occurs after a stroke that impacts spoken language expression, written expression, spoken language comprehension, and reading comprehension (Oliveira et al., 2023). Estimates suggest that 18.4% of stroke survivors discharged from US hospitals have aphasia and the presence of aphasia is associated with lower functional stroke outcomes, longer stays in rehabilitation settings, and increased mortality (Ellis et al., 2018). Similar to general stroke outcomes, people with aphasia (PWA) are left with reductions in functional communication that negatively impact their life satisfaction and quality of life (Lazar, 2017; Ellis, 2017).

Recent studies have also shown that sex differences exist in rates of aphasia (Armour et al., 2019; Wallentin, 2018). Wallentin et al. completed a meta-analysis of rates of aphasia and found that women had a significantly higher risk of aphasia than men (1.1-1.14 ratio) (Armour et al., 2019). Similarly, in a second meta-analysis, Li et al. (2024) found that the risk of aphasia was 1.23 times higher in women compared to men (Wallentin, 2018). The study showed that the prevalence of aphasia after stroke was 36% in women compared to 31% in men. According to Ellis et al. (2017) a number of factors potentially contribute to differences in rates of aphasia, including the sample sizes of stroke survivors included in prior studies, measurement approaches designed to identify aphasia, and geographic location (Li et al., 2024).

Understanding where the rehabilitation and specifically speech-language pathology (SLP) services are initiated for PWA and their discharge disposition is important to understanding aphasia-related outcomes and potential sex differences. Best practice recommendations for aphasia suggest PWA should receive services beginning early during the acute stage of their stroke and continue during the recovery process (Simmons-Mackie et al., 2017; Wallace et al., 2024; Burton et al., 2023). For example, stroke survivors should be screened for aphasia, and those identified should receive additional comprehensive evaluations to determine the nature and severity of their condition (Simmons-Mackie et al., 2017). Families of PWA should also be involved to ensure communication among patients, providers, and families concerning their condition and next steps (Simmons-Mackie et al., 2017; Wallace et al., 2024; Burton et al., 2023). Finally, PWA and their families should receive tailored training before they are discharged home to facilitate greater communication (Burton et al., 2023).

The objective of this study was to characterize sex differences in the location of initial aphasia diagnosis and the discharge destination of fee-for-service (FFS) Medicare beneficiaries diagnosed with stroke between 2016 and 2019. Understanding potential where services are initiated, continued during their recovery, and the environments they are discharged to is too important to understand overall sex differences in aphasia outcomes. Finally, understanding where services are initiated and where PWA are discharged to is ultimately important to constantly evaluating the aphasia management process and continuing to improve aphasia outcomes.

## Methods

This study was reviewed and approved by the University of Florida Institutional Review Board as an exempt study, #IRB202300891 due to its use of existing, de-identified data that could not be linked back to individualsubjects.

### Data source

Data for this project comprised a 100% sample of Medicare claims filed between 2016 and 2019. The Medicare Master Beneficiary Summary File (MBSF) provided information on beneficiary characteristics, including age, race, sex, and entitlement codes. Claims included coverage for Outpatient, Skilled Nursing Facility (SNF), Home Health Agency (HHA), Inpatient, and Carrier files. Outpatient claims covered services from hospital outpatient departments, clinics, and ambulatory surgical centers. SNF claims include post-acute care, therapy, rehabilitation, and nursing services. HHA claims covered skilled home health care services, including nursing, physical, occupational, and speech therapy. Inpatient claims involved short-term acute care, long-term acute care, inpatient rehabilitation, and inpatient psychiatric care. Carrier files consisted of data originating from individual practitioners or supplier organizations, rather than hospitals or other institutional facilities.

To identify the setting of aphasia diagnosis, the first Medicare claim containing an aphasia diagnosis code was identified for each stroke survivor. The location of service was then determined using claim-type–specific indicators: for inpatient claims, the last four digits of the CMS Certification Number (CCN) were used to classify facility type (see Appendix); for carrier claims, the place of service (POS) code was used to identify the setting in which care was rendered. Each POS code corresponds to a specific type of service location, such as a physician's office (POS 11), inpatient hospital (POS 21), outpatient hospital (POS 22), skilled nursing facility (POS 31), or home (POS 12).

### Sample

The index hospitalization was defined as an acute care hospital admission for a first-time ischemic or hemorrhagic stroke. The presence of stroke was identified from inpatient claims listing stroke as the primary diagnosis between January 1, 2016, and October 1, 2019. While traditional Medicare operates transparently through a claim-based reimbursement process, allowing all care, providers, and diagnoses to be viewed within the claims, Medicare Advantage functions under a capitated payment system. Under this system, private insurance companies receive a fixed monthly payment per enrollee to cover all necessary healthcare services. Since these private insurers manage care internally, they do not submit detailed claims to Medicare. Medicare collects encounter data only from MA plans that summarize services provided rather than detailing each claim. Therefore, all beneficiaries not enrolled in traditional fee-for-service (FFS) Medicare for 12 months were excluded. Post-stroke aphasia was identified for beneficiaries with a diagnosis code (aphasia R47.01, I69.320, I69.920) in the IP, OP, Carrier, HHA, or SNF claims files filed within 3 months (90 days) of the index hospitalization.

### Analysis

To characterize the first-time stroke and aphasia samples, frequency values for beneficiary-level age, sex, entitlement group, discharge disposition, and location of first aphasia claim were calculated. Given the proven validity of the Research Triangle Institute racial classification, the RTI race variable was used for classification. Entitlement codes included Old Age and Survivors Insurance (OASI), Disability Insurance Benefits (DIB), End-stage Renal Disease (ESRD), Both DIB and ESRD, and Beneficiary Insured Due to Part B Immunosuppressive Drug (PBID). Due to CMS confidentiality restrictions that prohibit reporting cell sizes below 11, DIB, ESRD, and DIB/ESRD were combined into a single category. Because Medicare has more than 40 discharge status codes, facility-level groupings were created. Finally, Charlson Comorbidity Index (CCI) values were calculated for all FFS beneficiaries using diagnoses listed in the OP, IP, and Carrier files following the methodology of Cenzer et al. (2013).

## Results

**Figure 1:**
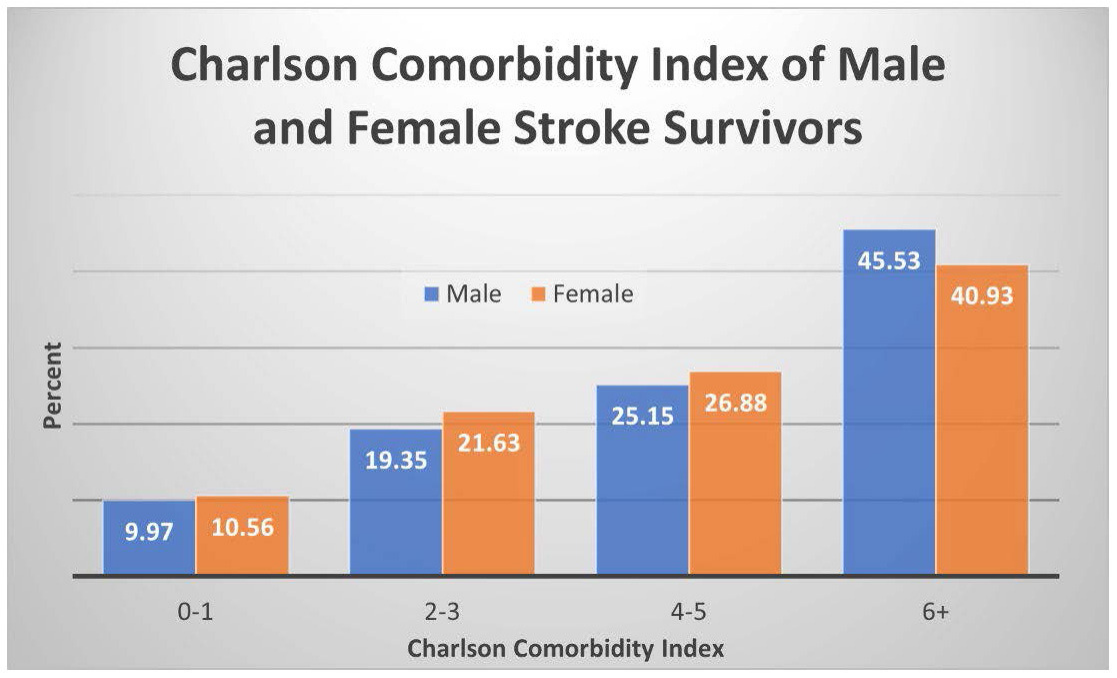
Charlson Comorbidity Index of male and female stroke survivors.

Characteristics of the Medicare fee-for-service beneficiaries with first-time stroke are listed in Table 1. These data reflect admissions between January 1, 2016, and October 1, 2019. Stroke survivors comprised 54.32% females and 45.68% males. Nearly 80% of females were non-Hispanic (NH) White, 12.47% were NH Black, 5.53% were Hispanic, and 4.05% were unknown, other, Asian/Pacific Islander, American Indian, and Alaska Native (UOAIAN). Among males, 77.81% were NH White, 11.38% were NH Black, 6.01% were Hispanic, and 4.81% were UOAIAN—a statistically significant difference (χ^2^=505.46, p,0.0001).Nearly 90% of males (89.16%) and 92.31% of female beneficiaries were over 65 and received Medicare through the OASI (Old-Age and Survivors Insurance) program (89.52% males, 93.3% females, χ^2^=2208.22, *p* < 0.0001). As shown in Figure 1, 45.53% of males had a CCI of 6 or higher, compared with 40.93% of females (χ^2^ = 1651.28, *p* < 0.0001). While 36.18% of men were discharged home, 17.14% to a SNF, and 20.66% to a long-term care (LTC) or rehabilitation facility, only 29.51% of women were discharged, 22.2% to a SNF, and 18.42% to a LTC or rehabilitation facility (χ^2^ =7654.27, *p* < 0.0001).

**Table 1:** Characteristics of Medicare fee-for-service stroke survivors.

		Male (*N* = 340,723, 45.68%)		Female (*N* = 405,194, 54.32%)		Sex difference	
Domain	Category	*N*	PCT	*N*	PCT	χ^2^	*p-*Value
Age Race/ Ethnicity	< 65	36,672	10.76	30,974	7.64	2,183.09 505.46	< 0.0001 < 0.0001
	> = 65	3,04,051	89.24	3,74,220	92.36		
	Non-Hispanic White	2,65,100	77.81	3,15,872	77.96		
	Black/African American	38,758	11.38	50,528	12.47		
	Hispanic	20,487	6.01	22,401	5.53		
	Unknown, Other, Asian/Pacific Islander, American Indian, Alaska Native	16,378	4.81	16,393	4.05		
Entitlement	OASI	30,3792	89.16	3,74,023	92.31	2,208.22	< 0.0001
	DIB, ESRD, DIB/ESRD, PBID	36,931	10.84	31,171	7.69		
Charlson comorbidity index	0-1	33,978	9.97	42,801	10.56	1,651.28	< 0.0001
	2-3	65,941	19.35	87,625	21.63		
	4-5	85,677	25.15	1,08,928	26.88		
	6 +	1,55,127	45.53	1,65,840	40.93		
Discharge Destination	Home	1,22,811	36.18	1,19,257	29.51	7,354.27	< 0.0001
	Transfer to Acute Care	7,243	2.13	7,046	1.74		
	SNF	58,200	17.14	89,711	22.2		
	LTC/Rehab	70,150	20.66	74,430	18.42		
	Home Health	37,370	11.01	54,778	13.55		
	Died	24,761	7.29	29,272	7.24		
	Hospice/Other Facility	18,943	5.58	29,663	7.34		

### Characteristics of people with aphasia

Table 2 presents the demographic, clinical, and service-use characteristics of Medicare fee-for-service beneficiaries with aphasia, stratified by sex. Of the 107,753 beneficiaries identified, 55.6% were female and 44.4% were male. Significant sex differences were observed in the age and race/ethnicity distributions (all *p* < 0.0001). A greater proportion of females were aged 65 years or older compared with males (93.3% vs. 89.6%), while males were more likely to be under age 65 (10.4% vs. 6.7%). The majority of beneficiaries in both groups were NH White (76.4% of males; 77.9% of females). Black/African American beneficiaries represented similar proportions among males and females (13.3% vs. 13.5%), whereas males were slightly more likely to be Hispanic or categorized as unknown/other race compared with females. Females were more likely to qualify for Medicare through Old-Age and Survivors Insurance (OASI) than males (93.3% vs. 89.5%), whereas males were more frequently entitled through disability-related pathways, including Disability Insurance Benefits and End-Stage Renal Disease (10.5% vs. 6.7%). Although distributions of the CCI were broadly similar, males were more likely to have the highest comorbidity burden (Charlson score ≥6; 54.0% vs. 49.6%), while females were more frequently represented in the moderate comorbidity categories (scores 2–5).

**Table 2:** Characteristics of Medicare fee-for-service people with aphasia.

		Male (*N* = 340,723, 45.68%)		Female (*N* = 405,194, 54.32%)		Sex difference	
Domain	Category	*N*	PCT	*N*	PCT	χ^2^	*p-*Value
Age	< 65	4,987	10.43	3,996	6.67	491.23	< 0.0001
	>=65	42,847	89.57	55,923	93.33		
Race/ Ethnicity	Non-Hispanic White	36,554	76.42	46,695	77.93	93.19	< 0.0001
	Black/African American	6,376	13.33	8,090	13.5		
	Hispanic	2,633	5.5	2,855	4.76		
	Unknown, Other, Asian/Pacific Islander, American Indian, Alaska Native	2,271	4.75	2,279	3.8		
Entitlement	OASI	42,820	89.52	55,906	93.3	496.39	< 0.0001
	DIB, ESRD, DIB/ESRD, PBID	5,014	10.48	4,013	6.7		
Charlson comorbidity index	0-1	3,006	6.28	4,075	6.8	208.31	< 0.0001
	2-3	6,916	14.46	9,765	16.3		
	4-5	12,100	25.3	16,364	27.31		
	6+	25,812	53.96	29,715	49.59		
Initial Service Identification	Inpatient facility	29,160	60.96	33,838	56.47	112.19	< 0.0001
	Rehabilitation facility	1,184	2.48	1,416	2.36		
	Skilled nursing facility	7,232	15.12	11,666	19.47		
	Home health	3,187	6.66	4,925	8.22		
	Outpatient facility	4,038	8.44	4,212	7.03		
	Office location	1,978	4.14	2,450	4.09		
	Ambulance/Other	1,055	2.21	1,412	2.36		
Discharge Destination	Home	7,137	14.99	6,865	11.5	904.70	< 0.0001
	Transfer to Acute Care	1,346	2.83	1,468	2.46		
	SNF	12,079	25.38	18,726	31.37		
	LTC/Rehab	20,439	42.94	22,823	38.24		
	Home Health	4,292	9.02	6,439	10.79		
	Died	951	2	1,070	1.79		
	Hospice/Other Facility	1,354	2.84	2,294	3.84		

### Initial aphasia diagnosis and discharge disposition

Although distributions were broadly similar, males were more likely to have the highest comorbidity burden (Charlson score ≥6; 54.0% vs. 49.6%, χ^2^ =208.31, *p* < 0.0001), while females were more frequently represented in the moderate comorbidity categories (scores 2–5). Males were more likely to be discharged home (15.0% vs. 11.5%) or to long-term care or rehabilitation facilities (42.9% vs. 38.2%), whereas females were more frequently discharged to skilled nursing facilities (31.4% vs. 25.4%) or with home health services (10.8% vs. 9.0%). Mortality during the index episode was low and similar between groups (2.0% of males vs. 1.8% of females).

As shown in Table 2, men and women differed significantly in the location of their initial aphasia diagnosis (χ^2^ = 112.19, *p* < 0.0001). Table 3 presents adjusted odds ratios (ORs) and 95% confidence intervals (CIs) from multivariable logistic regression models estimating the relative likelihood of (1) aphasia being first identified in an inpatient setting, (2) discharge to a skilled nursing facility (SNF), and (3) discharge to a long-term care (LTC) or rehabilitation facility, relative to reference groups.

**Table 3:** Relative likelihood of discharge disposition and identification location of FFS medicare beneficiaries with aphasia.

Domain	Estimate	Std err	Chi-square	Pr > ChiSq	Odds ratio	95% Confidence interval	
Aphasia identified in inpatient setting							
Intercept	**−0.62**	0.02	1515.02	< 0.0001			
Age < 65	0.17	0.13	1.87	0.17	1.42	0.86	2.33
CCI 2-3	**−0.12**	0.02	64.70	< 0.0001	0.96	0.90	1.02
CCI 4-5	**0.05**	0.01	18.12	< 0.0001	1.14	1.08	1.21
CCI 6+	**0.15**	0.01	179.61	< 0.0001	1.25	1.19	1.32
Black/African American	**0.06**	0.02	12.71	0.00	1.25	1.21	1.30
Hispanic	**0.13**	0.02	30.51	< 0.0001	1.34	1.26	1.42
Unknown, Other, Asian/Pacific Islander, American Indian, Alaska Native	**–**0.02	0.02	0.74	0.39	1.15	1.08	1.23
Female	**−0.08**	0.01	154.11	< 0.0001	0.85	0.83	0.87
Entitlement DIB, ESRD, DIB/ESRD, PBID	**–**0.09	0.13	0.52	0.47	0.83	0.51	1.37
Discharged to a skilled nursing facility							
Intercept	**−1.17**	0.02	4424.22	< 0.0001			
Age < 65	0.11	0.14	0.55	0.46	1.24	0.71	2.17
CCI 2-3	**−0.18**	0.02	128.24	< 0.0001	0.94	0.88	1.00
CCI 4-5	**0.05**	0.01	11.67	0.00	1.17	1.11	1.25
CCI 6 +	**0.25**	0.01	488.39	< 0.0001	1.44	1.36	1.53
Black/African American	**0.13**	0.02	51.61	< 0.0001	1.18	1.13	1.23
Hispanic	**−0.06**	0.02	5.83	0.02	0.98	0.92	1.04
Unknown, Other, Asian/Pacific Islander, American Indian, Alaska Native	**–**0.03	0.03	1.43	0.23	1.01	0.94	1.08
Female	**0.15**	0.01	472.32	< 0.0001	1.35	1.31	1.39
Entitlement DIB, ESRD, DIB/ESRD, PBID	**–**0.27	0.14	3.59	0.06	0.58	0.33	1.02
Discharged to a LTC/rehabilitation facility							
Intercept	**−0.49**	0.02	1010.48	< 0.0001			
Age < 65	0.10	0.12	0.62	0.43	1.21	0.75	1.95
CCI 2-3	**−0.07**	0.01	20.93	< 0.0001	1.34	1.26	1.42
CCI 4-5	**0.19**	0.01	239.82	< 0.0001	1.72	1.63	1.83
CCI 6+	**0.24**	0.01	501.63	< 0.0001	1.81	1.72	1.91
Black/African American	0.01	0.02	0.14	0.71	1.01	0.97	1.04
Hispanic	0.03	0.02	1.47	0.23	1.03	0.97	1.09
Unknown, Other, Asian/Pacific Islander, American Indian, Alaska Native	**–**0.03	0.02	1.97	0.16	0.97	0.91	1.03
Female	**−0.09**	0.01	207.83	< 0.0001	0.83	0.81	0.86
Entitlement DIB, ESRD, DIB/ESRD, PBID	**–**0.05	0.12	0.17	0.68	0.91	0.56	1.46
Reference Group: Age (≥65), CCI (0-1), Race/Ethnicity (NH White), Sex (Male), Entitlement (OASI).

Bold values indicate significant values.

After adjustment, higher comorbidity burden was strongly associated with increased odds of aphasia being first identified in an inpatient setting. Compared with beneficiaries with a Charlson Comorbidity Index (CCI) score of 0–1, those with CCI scores of 4–5 (OR = 1.14; 95% CI: 1.08–1.21) and ≥6 (OR = 1.25; 95% CI: 1.19–1.32) had higher odds of inpatient identification. Race and ethnicity were also associated with inpatient identification: Black/African American beneficiaries (OR = 1.25; 95% CI: 1.21–1.30) and Hispanic beneficiaries (OR = 1.34; 95% CI: 1.26–1.42) had higher odds compared with non-Hispanic White beneficiaries. Female beneficiaries had lower odds of aphasia being identified in an inpatient setting compared with males (OR = 0.85; 95% CI: 0.83–0.87). Age under 65 and disability-based Medicare entitlement were not significantly associated with inpatient identification.

Greater comorbidity burden was associated with increased odds of discharge to a skilled nursing facility. Relative to beneficiaries with CCI scores of 0-1, those with CCI scores of 4-5 (OR = 1.17; 95% CI: 1.11-1.25) and ≥6 (OR = 1.44; 95% CI: 1.36-1.53) had higher odds of SNF discharge. Black/African American beneficiaries were more likely than non-Hispanic White beneficiaries to be discharged to an SNF (OR = 1.18; 95% CI: 1.13–1.23). Female beneficiaries had substantially higher odds of SNF discharge compared with males (OR = 1.35; 95% CI: 1.31–1.39). Age under 65, Hispanic ethnicity, and disability-based entitlement were not significantly associated with SNF discharge.

Increasing comorbidity burden was strongly associated with discharge to a long-term care or rehabilitation facility. Compared with beneficiaries with CCI scores of 0–1, those with CCI scores of 2–3 (OR = 1.34; 95% CI: 1.26–1.42), 4–5 (OR = 1.72; 95% CI: 1.63–1.83), and ≥6 (OR = 1.81; 95% CI: 1.72-1.91) had progressively higher odds of discharge to LTC/rehabilitation settings. Female beneficiaries had lower odds of discharge to LTC/rehabilitation compared with males (OR = 0.83; 95% CI: 0.81–0.86). Race/ethnicity, age under 65, and disability-based entitlement were not significantly associated with discharge to LTC/rehabilitation facilities.

## Discussion

In this study of over 100,000 Medicare beneficiaries who experienced a stroke and were subsequently diagnosed with aphasia, men were 15% more likely than women to have their initial aphasia diagnosis in an inpatient hospital setting. Similarly, men were more likely than women to have their initial aphasia diagnosis in an outpatient setting. In contrast, women were more likely to have their initial aphasia diagnosis in skilled nursing facilities when compared to men. Regarding discharge disposition, men were more likely to be discharged home, to long-term care, or to a rehabilitation facility than women. Similar to the initial encounter, women were more likely than men to be discharged to a skilled nursing facility. Interestingly, the issues related to where the initial aphasia diagnosis occurred, and their discharge disposition are interrelated.

Regardless of where women received their initial aphasia diagnosis, the number of women receiving their initial aphasia diagnosis was < 50%. For both men and women, the roughly 50% of PWA who did receive their initial encounter during their inpatient stay is concerning. These findings suggest that despite clinical practice guidelines for PWA calling for early identification and subsequent aphasia management approaches (Simmons-Mackie et al., 2017; Wallace et al., 2024; Burton et al., 2023), many are not receiving such care. These findings also suggest these issues are magnified among women with aphasia. They also add support to research that suggests women with stroke are less likely to received evidenced based care known to improve stroke-related outcomes when compared to men (Strong et al., 2020; Xu et al., 2025).

Regarding discharge disposition, the findings here align with previous observations in the stroke literature that show women are more likely to be discharged to nursing homes or SNFs than men. Evidence suggests women are more likely to be discharged from SNFs than men for a range of reasons, including older age, higher number of comorbid conditions, greater levels of disability, and higher levels of care needed during their recovery (Hayes et al., 2023; Pattath et al., 2023). Women discharged to SNFs also tended to have longer length of hospital stays, reflecting greater post-stroke disability and less ability to engage in high-intensity rehabilitation that frequently occurs within inpatient rehabilitation facilities (Pattath et al., 2023).

In addition, women were more likely to be admitted to SNFs because of a greater need for medical care than men for conditions such as pain and depression (Yu et al., 2020). Consequently, the impact of traditional pathways for stroke survivors had a similar impact on initial encounters by SLPs and their discharge settings. More specifically, if women are more likely to be discharged to SNFs, the impact of when they are first seen by SLPs would be a similar impact. Our own work has shown that while aphasia is a common condition after stroke, short hospital stays in combination with the differential characteristics of women compared to men (older age, greater disability, etc.) have resulted in many individuals not being identified early in the stroke recovery process [(Ellis and Jacobs, 2025)]. Other factors, such as being at larger hospitals and being at a hospital with a stroke unit, also seem to impact decisions regarding discharge to an SNF vs IRF, which in turn influence when initial encounters occur if they do not occur in acute care settings (Pattath et al., 2023).

Despite these interesting findings, this study has a number of limitations. Use of Medicare claims data has limitations that must be carefully considered. The use of secondary data, such as Medicare claims data used primarily for billing, can be influenced by coding inaccuracies (Cohen, 2014). In addition, Medicare claims data does not include important behavioral information related to stroke risk, nor does it include tests that are used to determine stroke severity (Mues et al., 2017). Finally, Medicare claims data is primarily related to individuals over the age of 65, which may not be representative of the overall stroke population (Mues et al., 2017).

In consideration of aphasia specifically, some claims utilized in this work come from multiple settings where services for PWA might differ somewhat. More specifically, inpatient claims may include any of the following: acute care hospitals, critical access hospitals, inpatient rehabilitation facilities, inpatient psychiatric facilities, and long-term care hospitals, all of which have very different missions. Finally, we noted that Medicare claims are very complex and require linkage across claims to identify conditions such as aphasia. The linkage process needed to capture these conditions can increase the likelihood of error and, in some cases, misinterpretation.

## Conclusions

Observed sex differences in this study should be carefully considered to reduce their potential contribution to disparities in stroke-related outcomes. Future research must be designed to better understand the cause of these observed differences and strategies to reduce the likelihood of their occurrence.

## Data Availability

The data analyzed in this study is subject to the following licenses/restrictions: Medicare data restrictions are governed by the Health Insurance Portability and Accountability Act (HIPAA) and strict Centers for Medicare & Medicaid Services (CMS) policies to protect Personally Identifiable Information (PII) and Protected Health Information (PHI). Requests to access these datasets should be directed to Medicare –https://www.cms.gov/about-cms/information-systems/privacy/data-use-agreement-dua.
