# Supplementary material for: Sex differences in location of initial diagnosis and discharge destination among medicare fee-for-service beneficiaries with post-stroke aphasia
Source: Front Stroke. 2026 Jul 29;5:1904901. doi: 10.3389/fstro.2026.1904901 (PMC13461540; doi:10.3389/fstro.2026.1904901)
Supplement: Supplementary file 1 [file Supplementary_file_1.pdf]

Appendix 1 – Codes Used to Identify Stroke

G45.0 G45.1 G45.2 G45.8 G45.9 G46.0 G46.1 G46.2 G46.3 G46.4 G46.5 G46.6 G46.7
G46.8 G97.31

G97.32 I60.00 I60.01 I60.02 I60.10 I60.11 I60.12 I60.20 I60.21 I60.22 I60.30 I60.31 I60.32
I60.4 I60.50

I60.51 I60.52 I60.6 I60.7 I60.8 I60.9 I61.0 I61.1 I61.2 I61.3 I61.4 I61.5 I61.6
I61.8 I61.9 I63.00 I63.011

I63.012 I63.013 I63.019 I63.02 I63.031 I63.032 I63.039 I63.09
I63.10 I63.111 I63.112 I63.113 I63.119

I63.12 I63.131 I63.132 I63.133 I63.139 I63.19 I63.20 I63.211
I63.212 I63.213 I63.219 I63.22 I63.231

I63.232 I63.233 I63.239 I63.29 I63.30 I63.311 I63.312 I63.313
I63.319 I63.321 I63.322 I63.323 I63.329

I63.331 I63.332 I63.333 I63.339 I63.341 I63.342 I63.343
I63.349 I63.39 I63.40 I63.411 I63.412 I63.413

I63.419 I63.421 I63.422 I63.423 I63.429 I63.431 I63.432
I63.433 I63.439 I63.441 I63.442 I63.443

I63.449 I63.49 I63.50 I63.511 I63.512 I63.513 I63.519 I63.521
I63.522 I63.523 I63.529 I63.531 I63.532

I63.533 I63.539 I63.541 I63.542 I63.543 I63.549 I63.59 I63.6
I63.8 I63.81 I63.89 I63.9 I66.01 I66.02

I66.03 I66.09 I66.11 I66.12 I66.13 I66.19 I66.21 I66.22 I66.23 I66.29 I66.3 I66.8 I66.9
I67.841 I67.848

I67.89 I97.810 I97.811 I97.820 I97.821

Appendix 2 – Last four digits of the CMS Certification Number (CCN) Used to Identify Setting of Aphasia Diagnosis

0001-0879	Short-term (General and Specialty) Hospitals
0880-0899	Reserved for hospitals participating in ORD demonstration project
0900-0999	Multiple Hospital Component in a Medical Complex (Numbers Retired)
1000-1199	Federally Qualified Health Centers
1200-1224	Alcohol/Drug Hospitals (Numbers Retired)
1225-1299	Medical Assistance Facilities
1300-1399	Critical Access Hospitals
1400-1499	Continuation of Community Mental Health Centers (4900-4999 series)
1500-1799	Hospices
1800-1989	Federally Qualified Health Centers
1990-1999	Religious Non-medical Health Care Institutions (formerly Christian Science Sanatoria (Hospital Services)
2000-2299	Long-Term Hospitals (Excluded from PPS)
2300-2499	Hospital Based Renal Dialysis Facilities
2500-2899	Independent Renal Dialysis Facilities
2900-2999	Independent Special Purpose Renal Dialysis Facility
3000-3024	Formerly Tuberculosis Hospitals (Numbers Retired)
3025-3099	Rehabilitation Hospitals (Excluded from PPS)
3100-3199	Home Health Agencies
3200-3299	Continuation of Comprehensive Outpatient Rehabilitation Facilities (4800-4899) Series
3300-3399	Children's Hospitals (Excluded from PPS)
3400-3499	Continuation of Rural Health Clinics (Provider-based) (3975-3999) Series
3500-3699	Hospital Based Satellite Renal Dialysis Facilities
3700-3799	Hospital Based Special Purpose Renal Dialysis Facility
3800-3974	Rural Health Clinics (Free-Standing)
3975-3999	Rural Health Clinics (Provider-Based)
4000-4499	Psychiatric Hospitals (Excluded from PPS)
4500-4599	Comprehensive Outpatient Rehabilitation Facilities
4600-4799	Community Mental Health Centers
4800-4899	Continuation of Comprehensive Outpatient Rehabilitation Facilities (4500-4599 Series)
4900-4999	Continuation of Community Mental Health Centers (4600-4799) Series
5000-6499	Skilled Nursing Facilities
6500-6989	Outpatient Physical Therapy Services
6990-6999	Numbers Reserved (formerly Christian Science Sanatoria (Skilled Nursing Services)
7000-8499	Continuation of Home Health Agencies (3100-3199) Series
8500-8899	Continuation of Rural Health Clinics (Provider-Based) (3400-3499) Series
8900-8999	Continuation of Rural Health Clinics (Free-Standing) (3800-3974) Series
9000-9799	Continuation of Home Health Agencies (8000-8499) Series
